# Screening for Residual Disease in Pediatric Burkitt Lymphoma Using Consensus Primer Pools

**DOI:** 10.1155/2009/412163

**Published:** 2009-04-15

**Authors:** Melissa Agsalda, Ian Kusao, David Troelstrup, Bruce Shiramizu

**Affiliations:** ^1^Department of Cell & Molecular Biology, John A. Burns School of Medicine, University of Hawaii, Honolulu, HI 96816, USA; ^2^Department of Physiology, John A. Burns School of Medicine, University of Hawaii, Honolulu, HI 96816, USA; ^3^Department of Pediatrics & Medicine, John A. Burns School of Medicine, University of Hawaii, Honolulu, HI 96816, USA

## Abstract

Assessing molecular persistent or minimal residual disease (PD/MRD) in childhood Burkitt lymphoma (BL) is challenging because access to original tumor is usually needed to design patient-specific primers (PSPs). Because BL is characterized by rearranged immunoglobulin heavy chain (IgV_H_) genes, IgV_H_ primer pools from IgV_H1_–IgV_H7_ regions were tested to detect PD/MRD, thus eliminating the need for original tumor. The focus of the current study was to assess the feasibility of using IgV_H_ primer pools to detect disease in clinical specimens. Fourteen children diagnosed with B-NHL had follow-up repository specimens available to assess PD/MRD. Of the 14 patients, 12 were PD/MRD negative after 2 months of therapy and remained in remission at the end of therapy; 2/14 patients were PD/MRD positive at 2-3 months and later relapsed. PSP-based assays from these 14 patients showed 100% concordance with the current assay. This feasibility study warrants further investigation to assess PD/MRD using IgV_H_ primer pools, which could have clinical significance as a real-time assessment tool to monitor pediatric BL and possibly other B-cell non-Hodgkin lymphoma therapy.

## 1. Introduction

The prognosis for children diagnosed with Burkitt lymphoma (BL) has improved dramatically over the last decade; however a significant percentage (30–40%) of children with advanced disease remain unresponsive to or relapse during therapy, particularly those with B-cell non-Hodgkin lymphoma (NHL) [1–3]. Because the prognosis for children who relapse during therapy is dismal, with an estimated 2-year overall survival of 10–30%, improvements in early detection of disease may improve their overall disease-free survival [2]. In such cases, identifying persistent disease or minimal residual disease (PD/MRD) could be important if the paradigm of early intervention when PD/MRD is found can be translated from other pediatric cancers such as acute lymphoblastic leukemia [4].

Malignant BL cells are usually characterized with immunoglobulin heavy chain variable region (IgV_H_) gene rearrangements. The IgV_H_ is formed during normal B-cell ontogeny by an ordered process of Ig gene rearrangement through assembling distinct variable (V), diversity (D), and joining (J) gene segments known as VDJ recombination [5]. A single V_H_ gene is chosen from the available V_H_ repertoire consisting of >50 potentially functional genes that are grouped into 7 structurally related families (V_H1–7_) [6]. Rearrangements of V_H_ genes can be exploited by analyzing the V_H1–7_ regions using polymerase chain reaction (PCR). We and others previously used this molecular fingerprint from NHL primary diagnostic tissue to detect PD/MRD in followup specimens [7–9]. However, as a clinical tool, identifying patient-specific primers (PSPs) designed from primary diagnostic tissue is labor-intensive and may not be applicable for real-time applications [7]. When faced with limited amount of tissue at the time of diagnosis, particularly from children, there may not be enough tissue available to design PSP if PD/MRD studies are to be tested [10–12]. The current study hypothesizes that detection of PD/MRD in BL cases may be accomplished by more efficient means and not require primary diagnostic tissue to design unique PSP. Instead, PD/MRD assessment could be achieved with primer pools made up of IgV_H_ oligomers from respective V_H1_ to V_H7_ families. Thus a strategy was designed to detect PD/MRD with consensus primer pools by real-time PCR [7]. We report data using the primer pool method and compare results previously reported by our lab using PSPs [13].

## 2. Methods and Materials

The study focused on the feasibility of using pools of oligomers comprised of primers from the seven V_H_ regions (V_H1–7_) to amplify a clonal immunoglobulin heavy chain rearrangement that would be characteristic of BL cells by real-time PCR. The study, which was approved by the Institutional Review Board, analyzed repository specimens and compared against previous results in which the specimens were characterized using PSPs.

### 2.1. Patients and Specimens

Fourteen children with Burkitt lymphoma (BL) and known clinical outcomes had specimens available which were previously analyzed using PSP [7]. The diagnoses and staging were confirmed through a central review mechanism [7]. DNA from primary diagnostic tumor tissues and staging/followup specimens (peripheral blood mononuclear cells, PBMCs; bone marrow aspirate, BMA; cerebrospinal fluid, CSF) were available from paraffin-embedded diagnostic tissue, unstained peripheral blood smears/bone marrow slides, and CSF cell pellets; see Table 1 [7]. DNA was assessed by ultraviolet spectrophotometry and by amplification with beta-globin primers, which showed adequate quantity and quality for PCR [13]. Specimens were from entry (diagnostic) and followup time points (1-month and 2-month postinduction chemotherapy); see Table 1. From the primary diagnostic tumor tissue, PSPs were designed and used to assess PD/MRD on followup specimens as previously reported [7].

### 2.2. Real-Time PCR Using V_H1_–V_H7_ Primer Pools and Sensitivity Assessment

**Figure 1:**
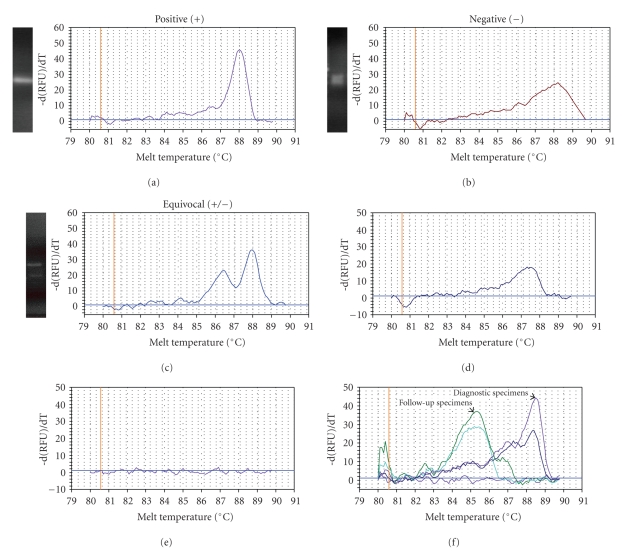
Examples of melt curve analyses with corresponding ethidium-stained amplified products. Melt curves displayed as −dF/dT plots: (a) defined peak with minimal to no shoulder indicates a positive MRD result; (b) a broad peak indicates a negative result; (c) a defined peak with significant shoulders indicates equivocal results. Representative 2.0% agarose gels are at the left of each of graph. Melt curves of negative controls: (d) PBMC from normal healthy individual shows a broad undefined peak; (e) water (no template) shows no peak; (f) melt curve assessment of PCR products: melt curve peak shift disparities are noted between diagnostic and followup specimens, consistent with possible different clonal differentiation over time.

Groups of 5′-primers from the FR1 region of the variable IgH regions, V_H_–V_H7_ (Operon Biotechnologies, Inc, Huntsville, AL, USA) were combined to form primer pools: V_H1_, V_H2_, V_H3_, V_H4_, V_H5_, V_H6_, and V_H7_ (Table 2) identified through VBASE23 (http://vbase.mrc-cpe.cam.ac.uk/) [14]. Primers were designed from the FR1 region because of future plans to use the assay on DNA extracted from fresh or unstained slides of PBMC, BMA, and CSF. The two consensus 3′-primers were: LJH and VLJH [7]. Seminested real-time PCR was performed in triplicate using an iCycler (BioRad Laboratories, Hercules, CA, USA), and StepOnePlus (Applied Biosystems, Foster City, CA, USA). The PCR parameters were optimized initially with DNA from a B-cell NHL cell line, Ramos (ATCC, Manassas, VA, USA) by diluting control human PBMC DNA as noted below with negative and positive controls consisting of peripheral blood mononuclear cells (PBMCs), water, and BL tumor DNA. The primer pool concentrations of 10 pmol were found to be optimal with the PCR mix of 2x iQ SYBR Green Supermix (Biorad Laboratories, Hercules, CA, USA), 10 pmol 5′-IgV_H_ primer pools and 10 pmol 3′-primer (LJH) and parameters: 95°C/3 minutes; 35 cycles 95°C/10 seconds, 60°C/30 seconds; 72°C/3 minutes; final extension of 72°C/3 minutes. PCR products were purified using ExoSAP-it (USB Corp, Cleveland, Ohio, USA), and recovered in 10 **μ**L. An aliquot of 1 **μ**L was used for second-round PCR with 10 pmol 5′-primer pools and 10 pmol 3′-primer (VLJH). Following threshold-dependent cycling, melting was performed from 60 to 95°C at either 0.5°C/s or 0.1°C/s melt rates with a smooth curve setting averaging 1 point with minor modifications of a previously described strategy to identify unique amplified products [9, 15]. Melt curves were plotted as the negative first derivative of decrease in fluorescence versus temperature (−dF/−dT). Polyclonal products of different lengths would melt at different temperatures and displayed as broad peaks. A wide melt curve peak defined as the width at half height ≥ peak height was considered a negative result modified from previous reports due to different primers and compensations made in the current real-time methodology versus the plate reader assay [16, 17]. Thus a positive (peak height ≥ width at half height); negative (peak height ≤ width at half height) or equivocal (multiple peaks) determination was established for each melt curve, (Figure 1) with verification by resolving the amplified products on agarose gels and sequencing, if appropriate. Differentiation by melt-curve analysis led to distinct melt temperatures for distinct amplified products if a unique V_H_ region is amplified and represented as a sharp peak [18, 19]. Following PCR, samples were subjected to two melt runs, with melt rates of either 0.1 or 0.5°C/s if melt peaks could not be distinguished [20]. As noted by others, increasing the melt rate can increase the size of the melt peaks due to a more rapid loss of fluorescence, facilitating more reliable detection of weaker amplicons [20]. Sharp melt peaks corresponded to monoclonal bands while widened or flat melt curves suggested polyclonal or no clonal DNA, Figures 1(b) and 1(c). If a melt curve suggested polyclonality, PCR products were purified and sequenced. For the current study, polyclonality determined by the sequence data was interpreted as negative for MRD/PD.

An algorithm was established to screen for V_H_ family usage. In this study, each primary tumor tissue DNA was subjected to each of the following combinations of primer pools: V_H1_ & V_H2_; V_H3_ & V_H4_; V_H5_, V_H6_, & V_H7_. The strategy allowed for combinations of V_H_ primers to be used to screen-in or screen-out involvement of variable regions. If particular primer pools were positive, then individual V_H_ primers were used to individually retest the tumor tissue DNA to identify the variable region involved. The specific V_H_ primer was then used to assess PD/MRD on sequential specimens from the same patient. Sensitivity of the assay was determined by two methods using cell line DNA and BL tumor DNA. DNA from the B-cell NHL Ramos cell line and control human PBMC were combined in various dilutions (DNA equivalent of 1 Ramos cell to 10^2^, 10^3^, 10^5^, 10^7^, 10^8^, and 10^9^ PBMC DNA) to test the IgV_H_ primer pools [21]. Dilutions of DNA from tumor DNA were prepared with control human PBMC to the DNA equivalent of 1 malignant cell to 10^3^, 10^5^, 10^6^, 10^8^, and 10^9^ PBMC DNA. Each dilution was then assayed using V_H4_ primer, which was a characteristic of that particular tumor specimen.

## 3. Results

### 3.1. Real-Time PCR Using V_H1_–V_H7_ Primer Pools and Sensitivity Assessment

**Figure 2:**
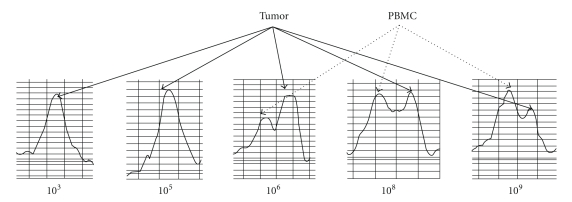
MRD sensitivity with patient primary tumor DNA. Primary diagnostic tumor DNA (100% infiltrated with malignant cells) was diluted with PBMC DNA to represent the equivalent of 1 malignant cell in 10^3^, 10^5^, 10^7^, 10^8^, and 10^9^ PBMC. Each representative melt curve has a distinct peak corresponding to tumor DNA or PBMC DNA, which are designated by the respective arrows.

Dilutions of DNA from the control Ramos cell line were assayed using the IgV_H_ primer pools [22]. Melt curve analysis of each reaction was plotted as the negative first derivative of decrease in fluorescence with respect to temperature (−dF/dT) and interpreted: Positive (distinct peak with minimal or no shoulder); Negative (broad peak); or Equivocal (multiple peaks with significant shoulder); see Figure 1. The dilution experiments demonstrated that Ramos cells could be detected on the order of 1 in 10^5^ to 1 in 10^6^ PBMC. Using patient tumor DNA, the sensitivity of the assay could detect malignant cells as low as 1 in 10^5^ PBMC (Figure 2).

PD/MRD assays were performed on clinical specimens from the 14 subjects in which primary diagnostic tissues were available. The results were compared to the analyses from the PSP method and found 100% concordance in that when PD/MRD was positive via PSP method, the same result was obtained using IgV_H_ primer pools (Table 1) [7]. The amplified products from the positive PD/MRD specimens were sequenced, confirming the monoclonality of the specimens.

Patients, NHL-5 and NHL-7, continued to have PD/MRD in followup BMA specimens 2 months after starting chemotherapy, and subsequently developed clinically detectable relapse at 6 months and 3 months, respectively. The amplified products from the BMA from these two subjects were purified and sequenced, which confirmed the same clone as the original primary diagnostic tumor. The other 12 cases demonstrated clearance of their followup blood and/or bone marrow specimens and at the end of therapy, remained in clinical remission. While no followup specimens were available from patients NHL-5 and NHL-7, the specimens which were positive for PD/MRD did not have evidence of disease by standard microscopic pathology.

To determine the utility of the methodology in assessing PD/MRD in specimens when primary diagnostic tissues were not available, staging (Entry) specimens from each of the cases were assayed using IgV_H_ primer pools using the same strategy noted above. Each subject had staging specimens which were positive for PD/MRD by a primer pool (Table 1). This was important to verify, to validate the use of the strategy in cases in which primary tumor tissue was not available.

## 4. Discussion

The current study was designed to assess PD/MRD on followup specimens from pediatric B-NHL cases regardless of whether primary diagnostic tissue was available from the time of diagnosis. We hypothesized that PD/MRD could be screened in specimens using primer pools made up of IgV_H_ oligomers from respective V_H1_ to V_H7_ families. The method was compared to a previous study, which used the PSP-based assay, and the results demonstrated 100% concordance [7]. Sequencing of the amplified products from primary diagnostic tumor DNA confirmed the results from the V_H_ oligomer amplified product demonstrating that monoclonal results were represented by both methods.

The use of IgV_H_ primer pools to screen for PD/MRD in clinical specimens was shown to have potential and feasibility. The current study is limited by the number of subjects and specimens available; however, as a feasibility study, the data support future consideration of the tool to determine the clinical significance of PD/MRD in pediatric BL and possibly B-cell NHL therapy trials. A potential limitation of the approach describe in this paper is that the BL clone needs to have rearrangement of an IgH gene [23]. Thus in BL cases involving immunoglobulin light chain rearrangements, the primer pools will need to be redesigned [23, 24]. There is precedence for potential utility of real-time assessment of PD/MRD. PCR-based MRD assays have been tested clinically in childhood acute lymphoblastic leukemia (ALL) by targeting IgH gene rearrangements with sensitivities on the order of one leukemic cell in 10^4^–10^6^ normal cells. Rapid reduction of MRD following induction therapy was associated with a 3-year relapse rate less than 5%, while children with detectable MRD had a 3-year relapse rate of 23%–75% [25]. In ALL, MRD was the most powerful prognostic factor of outcome, independent of other risk factors (age, leukocyte count, cytogenetic abnormalities, prednisone response), with similar results in children undergoing BM transplantation [26, 27]. To date, translating the utility of MRD assessment in ALL to pediatric lymphoma has been challenging, in part due to access to primary diagnostic tissue in order to identify PSPs as a marker to be used to assess followup specimens. Other challenges include presence or absence of cytogenetic abnormalities such as 13q^−^ and 7q^+^, which could be useful for prognosis, but there remains a lack of consistent cytogenetic abnormalities other than *c-myc* in childhood NHL for use in FISH or PCR [28]. Thus, the current study could potentially be applicable in assessing PD/MRD through IgV_H_ oligomer pools in a PCR assay that does not rely on primary diagnostic tissue for a specific tumor marker. However there are currently no universally accepted or recognized standard methods to assess PD/MRD in pediatric BL for which to compare. Because the current study is preliminary, emphasis should be placed on the fact that the current assay cannot substitute for the use of PSP-based assay and in the absence of primary diagnostic tissue until further studies are completed.

Other investigators have studied the IgV_H_ region in NHL and found preferential V_H_ gene usage with V_H3_ and V_H4_ families [8]. For the current study, we screened the primary diagnostic specimens for all of the V_H_ gene families (V_H1_ to V_H7_) with the V_H_ primer sets and found that, for the limited number of cases, the majority included V_H3_ or V_H4_ families. The data suggest that the PCR assay using IgV_H_ primer pools is able to identify B-NHL DNA in clinical specimens, with the limitations as noted above. Clinical translational studies with larger number of patients are needed to assess the utility of this assay to detect PD/MRD and determine the clinical significance. If the assay is validated in larger studies, one of the potential advantages of the tool would be the ease of assessing PD/MRD without the need of primary diagnostic tissue.

## Figures and Tables

**Table 1:** Persistent or minimal residual disease assessment using real-time and PSP assays.

Case (Dx)	Real-time results/PSP results & clinical status*				
	Entry	1 Mo	2 Mo	3-4 Mo	Clinical status
NHL-1 (BL)	Tissue (+/PSP)				CR (End of Rx))
	Blood (+/+)	Blood (−/−)	Blood (+/+)	Blood (−/−)	

NHL-2 (BL)	Tissue (+/PSP)				CR (End of Rx)
	Blood (+/+)	Blood (+/+)		Blood (−/−)	
	BMA (+/+)				

NHL-3 (BL)	Tissue (+/PSP)				Dead (Infection; 3 mo)
	Blood (+/+)	Blood (+/+)			
	BMA (+/+)		BMA (−/−)		

NHL-4 (BL)	Tissue (+/PSP)				CR ( End of Rx )
	Blood (+/+)	Blood (+/+)	Blood (−/−)		

NHL-5 (BL)	Tissue (+/PSP)				BMA relapse (6 mo)
	Blood (+/+)	Blood (−/−)			
	BMA (+/+)	BMA (+/+)			

NHL-6 (BL)	Tissue (+/PSP)				CR ( End of Rx )
	BMA (+/+)	BMA (−/−)			

NHL-7 (BL)	Tissue (+/PSP)				BMA relapse (3 mo)
	BMA (+/+)	BMA (+/+)			

NHL-8 (BL)	Tissue (+/PSP)				
	Blood (+/+)	Blood (−/−)	Blood (−/−)		CR (End of Rx)

NHL-9 (BL)	Tissue (+/PSP)				
	Blood (+/+)	Blood (+/+)		Blood (−/−)	CR (End of Rx)
	CSF (+/+)			CSF (=/=)	

NHL-10 (BL)	Tissue (+/PSP)				
	Blood (+/+)			Blood (−/−)	CR (End of Rx)

NHL-11 (BL)	Tissue (+/PSP)				
	Blood (+/+)	Blood (+/+)	Blood (+/+)	Blood (−/−)	CR (End of Rx)

NHL-12 (BL)	Tissue (+/PSP)				
	Blood (+/+)	Blood (+/+)	Blood (−/−)		CR (End of Rx)
	BMA (Equiv)	BMA (+/+)	BMA (−/−)		
	CSF (+/+)				

NHL-13 (BL)	Tissue (+/PSP)				
	Blood (+/+)	Blood (=/=)	Blood (=/=)		
	BMA (+/+)			BMA(−/−)	CR (End of Rx)
				CSF(−/−)	

NHL-14 (BL)	Tissue (+/PSP)				
	Blood (+/+)	Blood (−/−)		Blood (−/−)	CR (End of Rx)
	BMA (+/+)				

*Specimens were not available from some time points; Dx: diagnosis; BL: Burkitt lymphoma; BMA: bone marrow aspirate; real-time results/patient-specific primer (PSPs) results: Positive (+), Negative (−), or Equivocal (=) for each assay result; CR: complete remission; End of Rx: End of therapy.

**Table 2:** Primers sequences.

Primer	Sequence (5′–3′)
VH1a	CAG GT(GT) CAG CTG GTG CAG
VH1b	CAG GTC CAG CTT GTG CAG
VH1c	(GC)AG GTC CAG CTG GTA CAG
VH1d	CA(AG) ATG CAG CTG GTG CAG
VH2a	CAG ATC ACC TTG AAG GAG
VH2b	CAG GTC ACC TTG A(AG)G GAG
VH3a	GA(AG) GTG CAG CTG GTG GAG
VH3b	CAG GTG CAG CTG GTG GAG
VH3c	GAG GTG CAG CTG TTG GAG
VH4a	CAG (CG)TG CAG CTG CAG GAG
VH4b	CAG (CG)TG CAG CTG CAG GAG
VH5a	GA(AG) GTG CAG CTG GTG CAG
VH6a	CAG GTA CAG CTG CAG CAG
VH7a	CAG GT(CG) CAG CTG GTG CAA
LJH	TGA GGA GAC GGT GAC C
VLJH	GTG ACC AGG GNC CTT GGC CCC AG
B-globin (forward)	GAA GAG CCA AGG ACA GGT AC
B-globin (reverse)	CAA CTT CAT CCA CGT TCA CC
